# Adiporon, an adiponectin receptor agonist acts as an antidepressant and metabolic regulator in a mouse model of depression

**DOI:** 10.1038/s41398-018-0210-y

**Published:** 2018-08-16

**Authors:** Sarah Nicolas, Delphine Debayle, Catherine Béchade, Luc Maroteaux, Anne-Sophie Gay, Pascale Bayer, Catherine Heurteaux, Alice Guyon, Joëlle Chabry

**Affiliations:** 1 Université Côte d’Azur, Institut de Pharmacologie Moléculaire et Cellulaire, UMR 7275 CNRS, 660 Route des lucioles, Sophia Antipolis 06560 Valbonne, France; 2 Physicochemical Characterization of Biomolecules CAPABIO platform, UMR 7275 CNRS, 660 Route des lucioles, Sophia Antipolis 06560 Valbonne, France; 3 Institut du Fer à Moulin UMR-S U839 Inserm, Université Pierre & Marie Curie, 17 Rue du Fer à Moulin, 75005 Paris, France; 4 Centre Hospitalier Universitaire de Nice, Hôpital Pasteur, Service de Biochimie, 30 Voie Romaine, 06000 Nice, France

## Abstract

Major depression is a psychiatric disorder with complex etiology. About 30% of depressive patients are resistant to antidepressants that are currently available, likely because they only target the monoaminergic systems. Thus, identification of novel antidepressants with a larger action spectrum is urgently required. Epidemiological data indicate high comorbidity between metabolic and psychiatric disorders, particularly obesity and depression. We used a well-characterized anxiety/depressive-like mouse model consisting of continuous input of corticosterone for seven consecutive weeks. A panel of reliable behavioral tests were conducted to assessing numerous facets of the depression-like state, including anxiety, resignation, reduced motivation, loss of pleasure, and social withdrawal. Furthermore, metabolic features including weight, adiposity, and plasma biological parameters (lipids, adipokines, and cytokines) were investigated in corticosterone-treated mice. Our data show that chronic administration of corticosterone induced the parallel onset of metabolic and behavioral dysfunctions in mice. AdipoRon, a potent adiponectin receptor agonist, prevented the corticosterone-induced early onset of moderate obesity and metabolic syndromes. Moreover, in all the behavioral tests, daily treatment with AdipoRon successfully reversed the corticosterone-induced depression-like state in mice. AdipoRon exerted its pleiotropic actions on various systems including hippocampal neurogenesis, serotonergic neurotransmission, neuroinflammation, and the tryptophan metabolic pathway, which can explain its antidepressant properties. Our study highlights the pivotal role of the adiponergic system in the development of both metabolic and psychiatric disorders. AdipoRon may constitute a promising novel antidepressant.

## Introduction

Major depression is a mood disorder with multifactorial origins, which affects about 350 million people worldwide. The symptomology is complex and characterized by sadness, low self-esteem, social withdrawal, loss of interest and pleasure, despair for several consecutive weeks, amongst other symptoms. Major depression can occur as a transient and isolated episode or in a chronic fashion. Current treatments are mainly based on monoaminergic system regulation such as selective serotonin reuptake inhibitors (SSRIs), and are readily available and efficient. However, about 30% of patients with depressive disorders are partially or completely resistant to treatments. Thus, there is an urgent need for novel therapeutic targets for the treatment of depression.

Beside monoaminergic system disturbances, depression has been linked to dysfunctions of other neuroendocrine and/or physiological functions, including the hypothalamo-pituitary-adrenal (HPA) axis. People with Cushing’s disease present a concomitant elevation in circulating cortisol and mood symptoms, suggesting that sustained elevation of cortisolemia is involved in the etiology of depression. The occurrence of depression increases after chronic corticosteroid therapy, and an elevated level of circulating cortisol has been observed in roughly half of people with major depression^1^. When corticosterone is administered over a long-term period to laboratory rodents, it causes several anxio-depressive symptoms including resignation, anhedonia, and reduced social interactions^2,3^.

Unregulated, elevated cortisol levels are linked with metabolic illnesses including obesity. In depressive patients, higher cortisolemia was found to correlate strictly with an increase in the abdominal fat mass^4,5^. Furthermore, epidemiological data indicate high comorbidity between metabolic and psychiatric disorders, particularly obesity and depression^6^. Obesity markedly increases the odds of developing depression^7–9^. Thus, depressive symptomology is closely linked to adipose-related metabolic signals including levels of glucocorticoid, adipokines, and cytokines, among others.

Interestingly, levels of the most abundant adipokine, adiponectin (ApN), have recently been associated with both depression and obesity and thus may represent a potential target against these disorders^9^. ApN was primarily involved in glucose and lipid metabolism^10,11^, and plasma ApN was found to negatively correlate with obesity and abdominal fat in humans^12,13^ and rodents^14,15^. Recent studies showed that ApN has a more widespread influence and functionality in the brain than previously thought^16^. Indeed, ApN regulates thermogenesis^17^, food intake^18^, and fear memory extinction^19^ through the activation of its membrane receptors AdipoR1 and AdipoR2, which are expressed throughout the brain. Patients with major depression displayed lower ApN plasma levels compared to controls^20,21^. Given that ApN knockout mice are more susceptible to anxio-depressive symptoms than wild-type (wt) mice, antidepressant-like properties have been ascribed to ApN^3,22,23^. Together, these data allow us to envisage the modulation of the adiponergic system as a possible therapy against depression related to metabolic dysfunction. Here, we show that AdipoRon, a non-peptidic agonist of ApN receptors^24^, rescues depression associated with moderate obesity and abdominal adiposity in an anxiety/depression-like mouse model induced by continuous input of glucocorticoids. Like classic antidepressants, AdipoRon promotes adult hippocampal neurogenesis, serotonin turnover, and serotonergic neurotransmission. Additionally, AdipoRon alleviates low-grade peripheral and central inflammation and regulates the Trp/L-Kyn pathway. Through its pleiotropic actions on multiple systems that are affected during depression, AdipoRon could constitute a powerful and promising antidepressant drug.

## Methods and materials

### Mice

Male C57BL/6J mice wild-type (wt) or transgenic ApN knock-out mice (ApN^−/−^) were obtained from Janvier (France) and the Jackson Laboratory, respectively. Mice were housed at 22 °C ± 1 on a 12-h light/dark cycle (lights on/off at 7 am/7 pm) and allowed free access to drink and chow (A04 Safe, 2900 kcal/kg).

### Chronic treatment of AdipoRon in a mouse model of depression-like behaviors

**Fig. 1:**
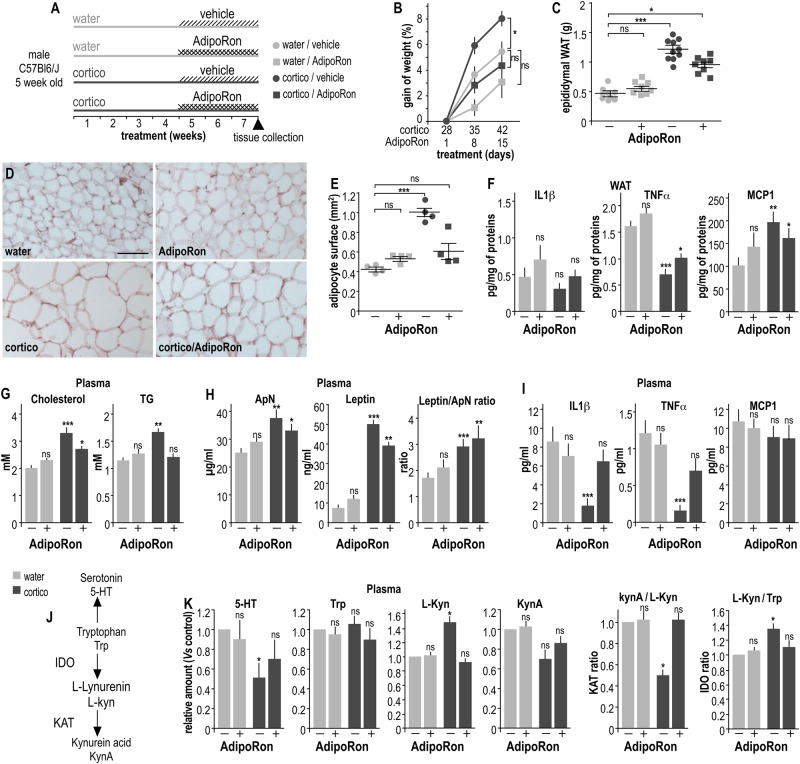
AdipoRon prevents excess weight gain, adiposity, and dyslipidemia in long-term corticosterone-treated mice. **a** Schematic representation of the experimental protocol. **b** Gain of weight expressed as percent of weight values at the onset of the AdipoRon or vehicle treatment from the four groups of mice: vehicle (light gray symbols), corticosterone (cortico, dark gray symbols), cortico with AdipoRon (1 mg/kg, squares) or cortico without AdipoRon (vehicle, circles). Values plotted are mean ± sem. A Friedman statistical analysis was followed by a Dunn’s post hoc test; *P < 0.05 (*N* = 10 per group). **c** Weights of epididymal adipose tissue from control (light gray) and cortico-treated (dark gray) mice chronically treated with vehicle (−) or 1 mg/kg of AdipoRon (+). **d** Representative images of eosin-hematoxylin-stained epididymal adipose tissue from control (water) and cortico-treated (cortico) mice with or without AdipoRon administration for 3 consecutive weeks. Scale bar, 50 µm. **e** Quantification of the surface area of adipocytes expressed in mm^2^, cells from five random fields containing about 100 cells/field were counted from four slices *per* mouse *per* group. Values plotted are mean ± sem; each symbol represents the mean of one mouse. **f** Concentrations of proinflammatory cytokines IL1β, TNFα, and chemokine MCP1 in epididymal fat tissue (expressed in pg/mg of total proteins) from control and cortico-treated mice ± AdipoRon administration. **g**–**i** Histograms showing the plasma concentrations of lipids (**g**; cholesterol, left; TG, right expressed in mM), adipokines (**h;** ApN, left, expressed in μg/ml; leptin, center, expressed in ng/ml; ratio leptin/ApN, right), and cytokines (**i**; IL1β, TNFα, and chemokine MCP1, expressed in pg/ml) from long-term cortico-treated mice ± AdipoRon administration. **j** Schematic representation of the Trp/Kyn pathway. **k** Histograms representing the relative plasma content of 5-HT, Trp, L-Lyn, KynA and the ratio KynA/L-Kyn and L-Kyn/Trp from control and cortico-treated mice ± AdipoRon administration. Experiments were conducted as described in SI-MM6. Means of data from vehicle-treated control mice were taken as 1. Values plotted are expressed as mean ± sem; *N* = 8–10 per group; Kruskal–Wallis statistical analysis was followed by a Dunn’s statistical test for comparison with the vehicle control group. ns non-significant; *P < 0.05, **P < 0.001, ***P < 0.001

**Fig. 2:**
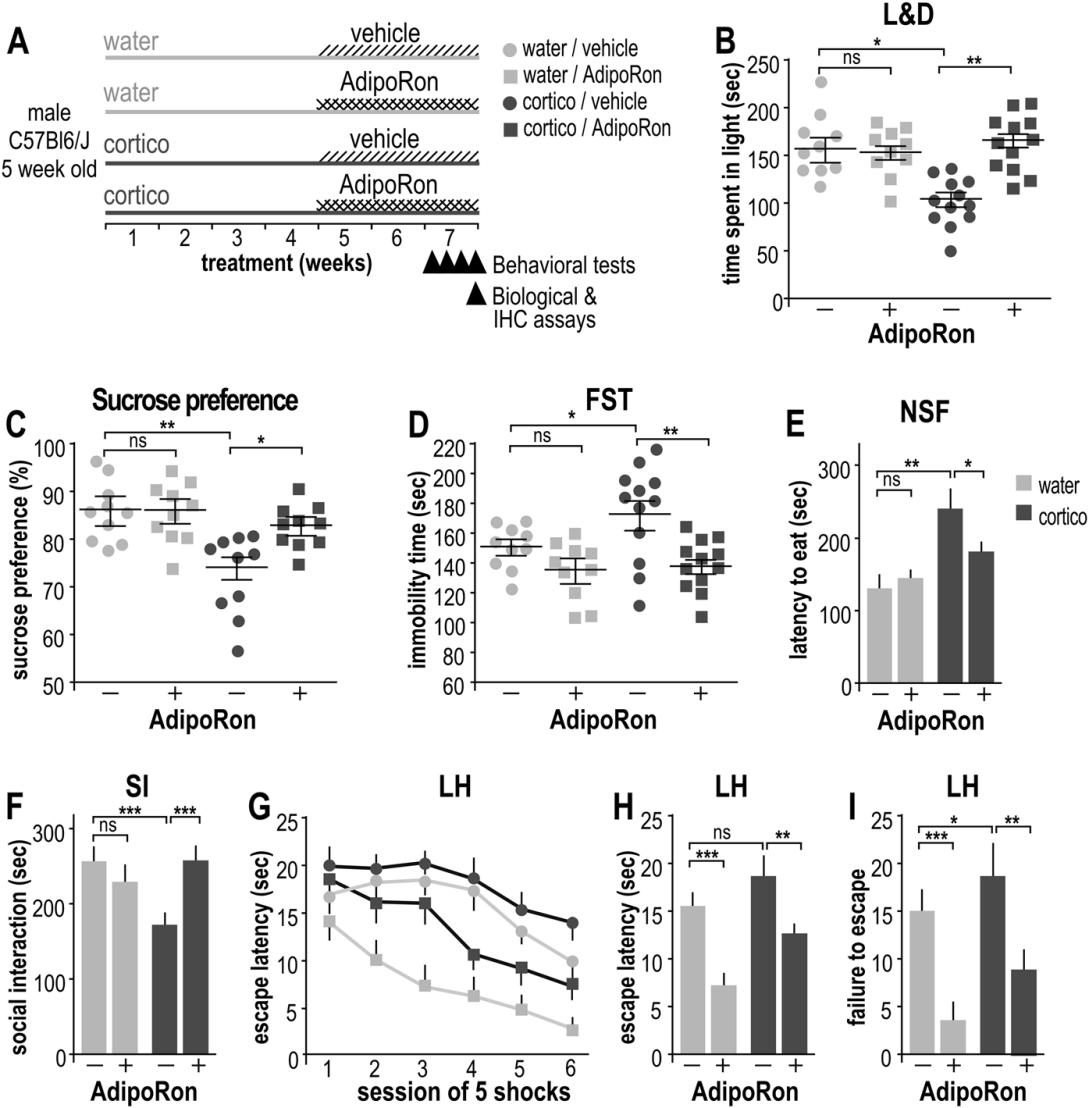
Antidepressant-like effects of AdipoRon assessed through behavioral tests on corticosterone-induced depressive-like mice. **a** Schematic representation of the experimental protocol (SI-MM1). Five-week-old male mice were randomly distributed to the following groups for a 7-week period, i.e., vehicle (light gray symbols), cortico (dark gray symbols), cortico + AdipoRon (1 mg/kg, squares) or cortico – AdipoRon (vehicle alone, circles). As described in the SI-MM1 and SI-MM2, during the seventh week of treatment, mice were submitted to behavioral testing: the light and dark test (**b**), the sucrose preference test (**c**), the FST (**d**), the NSF test (**e**), the social interaction test (**f**) and the learned helplessness test from which latency data were extracted (**g**, **h**) and the number of failures to escape the aversive box (**i**). Values plotted were mean ± sem; each symbol represents a mouse (*N* = 10–12 per group); Kruskal–Wallis statistical analysis was followed by a Dunn’s statistical test for comparison between groups. ns non-significant; *P < 0.05; **P < 0.01; ***P < 0.001

In the present study, the well-established mouse model of depression-like behavior based on the long-term corticosterone (cortico) treatment has been used^2^. Five-week-old wt male mice (five/cage) received cortico (35 mg/L dissolved in tap water containing 4 g/L βCD) or vehicle alone *ad libitum* for 7 consecutive weeks^2^. Cortico and vehicle solutions were changed every 3 days to prevent possible degradation. Additionally, during the last three weeks of the treatment, mice were intraperitoneally (*ip*) injected daily with AdipoRon (1 mg/kg in 2.5% DMSO) or vehicle alone (Figs. 1a and 2a and SI-MM1).

### Behavioral testing

All behavioral tests were conducted during the light phase (from 09:00 to 13:00) in compliance with the Institutional Animal Care and Use Committee of the University of Nice-Sophia Antipolis (permission number 010344.01 from the French “*Ministère de l’Enseignement Supérieur et de la Recherche*”). All data were obtained in blind-coded experiments, in which the investigators who obtained the data were unaware of the specific genotypes and treatments of mice (SI-MM2).

### Hippocampal neurogenesis and neuronal survival

To assay newborn hippocampal neuron survival and maturation, mice were *ip* injected with BrdU (50 mg/kg of body weight) once a day for five consecutive days, one week prior to the start of the chronic treatment with AdipoRon. For neurogenesis experiments, mice were *ip* injected with BrdU (50 mg/kg of body weight) three times, 3 h apart one day before the end of AdipoRon treatment. Either one day or 21 days after the last BrdU injections, mice were euthanized and transcardially perfused with cold PBS then fixed with 3.2% PFA. Brain sections were prepared for immunohistochemistry (IHC) as detailed in SI-MM3.

### Kinetic studies of Adiporon effects

Twelve-week-old male C57BL/6J mice were *ip* injected with AdipoRon (1 mg/kg) or vehicle alone (2.5% DMSO). At different time points after injection (0, 30 min, 1 h, 3 h, 6 h), mice were anesthetized by *ip* injection of sodium pentobarbital; blood samples were collected then perfusions were performed by intra-cardiac puncture of 30 ml of cold saline solution followed by dissection of various brain regions. Samples were stored at −80 °C until use.

### Brain tissue collection

Mice were deeply anesthetized with pentobarbital, and then perfused by intracardiac puncture of cold saline solution. Brains were collected and immediately cut into 1 mm thick slices with an ice-cold brain slicer matrix. Brain regions of interest were punched out, weighed, and stored at −80 °C.

### Cytokine measurements

Hypothalamus, hippocampus, prefrontal cortex, and epididymal white adipose tissue (WAT) samples were homogenized in lysis buffer (10 mM Tris-HCl, pH 7.5, 150 mM NaCl, 5 mM EDTA containing 10% glycerol and 1% NP-40 with protease inhibitor). MCP1, IFN-γ, IL-1β, IL-6, and TNFα levels were measured using an electrochemoluminescence (ECL)-based assay (U-plex, MesoScale Discovery, Rockville, MD, USA) following manufacturer instructions. Results were standardized according to the protein concentration of each sample as determined by the Bradford method.

### Triglyceride and cholesterol measurements

Plasma concentrations of cholesterol and triglycerides (TG) were assessed with an enzymatic colorimetric method (Kits from Roche diagnostic, respectively TRIGL and CHOL2) and on a “Roche Cobas c701” chemistry system.

### Statistical analysis

Statistical analyses were performed using GraphPad Prism 4.0 software. The Shapiro–Wilk test was systematically used to check for the normal distribution of the data. When the sample size was small (*n* ≤ 12) and/or in case of non-normal distribution, differences between more than two groups were assessed by the nonparametric Kruskal–Wallis test followed by a post hoc Dunn’s for multiple comparisons. Differences between two conditions were assessed by the nonparametric Mann–Whitney test. The nonparametric Friedman test was used when comparing groups of mice with repeated measurements (e.g., Figs. 1b and 2g). Data are presented as means ± standard error of the mean (sem); statistical significance was set at *P < 0.05 and **P < 0.01, ***P < 0.001.

## Results

### Chronic AdipoRon treatment ameliorates corticosterone-induced excess weight and dyslipidemia

We investigated the metabolic changes that occurred in cortico-induced depressive-like mice and the involvement of ApN pathway activation as assessed by chronic administration of the ApN agonist AdipoRon (Fig. 1a). Long-term treatment with cortico resulted in a significant weight gain in mice from the sixth week (Fig. 1b). The epididymal WAT mass (Fig. 1c) and the adipocyte surface area (Fig. 1d, e) were significantly increased in cortico-treated mice compared to controls. Interestingly, daily administration of AdipoRon during the last three weeks limited the corticosterone-induced increases of weight gain, epididymal mass, and adipocyte surface area, without any significant effect on control mice (Fig. 1b-e). The inflammation state of epididymal fat tissue was further investigated by measuring the in situ production of proinflammatory cytokines. Compared to the control group, WAT from the cortico-treated mice produced significantly less TNFα and more MCP1, independently of the AdipoRon treatment (Fig. 1f). None of the treatments significantly altered IL1β production in WAT (Fig. 1f). Long-term cortico-treatment resulted in elevated plasma levels of both cholesterol and TG compared to that of control mice, while AdipoRon prevented the cortico-induced increase of plasma lipids (Fig. 1g). The plasma concentrations of ApN and leptin were increased in cortico-treated mice compared to that of control group, but chronic administration of AdipoRon did not counteract this cortico-induced effect (Fig. 1h). The leptin/ApN ratio is considered to be a reliable indicator of metabolism dysfunctions and was increased in cortico-treated mice, independently of the AdipoRon treatment (Fig. 1h). Plasma concentrations of proinflammatory cytokines IL1β and TNFα were significantly reduced in the presence of cortico-treatment, while AdipoRon restored physiological levels (Fig. 1i). No significant variation in plasma concentrations of the chemokine MCP1 was observed, regardless of the treatment (Fig. 1i). The plasma and WAT concentrations of IL6 were virtually undetectable in all groups of mice (data not shown).

The Trp/L-Kyn pathway is markedly altered in overweight/obese patients and in rodent models of metabolic syndrome. Plasma levels of 5-HT, Trp, L-Kyn, and KynA were assayed by chromatography coupled to tandem mass spectrometry, allowing for the assessment of indoleamine 2,3-dioxygenase (IDO) and kynurenine aminotransferase (KAT) activities by means of the Trp/L-Kyn and KynA/L-Kyn ratios, respectively (Fig. 1j). Long-term administration of cortico triggered an increase in the plasma concentration of L-Kyn, resulting in a KynA/L-Kyn ratio decrease and a concomitant increase in L-Kyn/Trp (Fig. 1k). Chronic AdipoRon treatment significantly counteracted this effect, restoring the physiological IDO and KAT index. The 5-HT concentration was found to be decreased in plasma samples from cortico-treated mice compared to control mice. This parameter was also normalized by the chronic administration of AdipoRon (Fig. 1k). Note that in control mice, AdipoRon alone (i.e., without cortico treatment) did not alter the Trp/L-Kyn pathway, the plasma levels of lipids, adipokines, and cytokines, or the WAT features (Fig. 1c-k).

### Antidepressant effects of AdipoRon on a depression-like mouse model

The potential anxiolytic and antidepressant properties of Adiporon were investigated using long-term cortico treatment of mice (Fig. 2a). Cortico-treated mice presented exacerbated anxiety compared to control mice as demonstrated by a reduction in time spent in the aversive light chamber of the light-dark paradigm (Fig. 2b). Chronic administration of AdipoRon reversed the anxiety phenotype in the cortico-treated group, without significant effect on vehicle-treated mice (Fig. 2b). Long-term cortico treatment promoted depression-like behavior in mice, as shown by behavioral tests for anhedonia (sucrose preference test), resignation (FST), learned helplessness (LH), anxiodepressive-like behavior (NSF), and social interactions. Briefly, long-term exposure to cortico reduced the sucrose preference (Fig. 2c), increased the duration of immobility in the FST (Fig. 2d), the latency to eat in the NSF test (Fig. 2e), and lowered social interactions (Fig. 2f). Interestingly, chronic administration of AdipoRon reversed the depressive-like behavior induced by long-term administration of cortico in all these paradigms, whereas no significant effect was observed in the control group. In the LH paradigm, the latency to escape remained unchanged while the number of failures to escape the aversive area was increased in cortico-treated mice compared to controls, suggesting that long-term cortico treatment favored a resignation-like behavior (Fig. 2g-i). Chronic administration of AdipoRon prevented the resignation-like behavior in both healthy and depressive-like mice (Fig. 2g-i).

Chronic treatments with cortico and/or AdipoRon did not significantly modify locomotion and exploration activities assessed by the total traveled distance and mean speed in the OF (Fig. S1A, B, respectively), nor they did significantly affect motor coordination as assessed using the rotarod (Fig. S1C). In conclusion, using a set of behavioral tests, we showed that AdipoRon, counteracted depressive-like behaviors in a well-established mouse model of depression. Thus, AdipoRon presented antidepressant-like potential.

Together, our data suggest that chronic administration of cortico induced metabolic and behavioral dysfunctions in parallel, and these dysfunctions were at least partially counteracted by AdipoRon.

### Effect of acute administration of AdipoRon on various depression-like mouse models

The FST is the gold standard rodent behavioral test for rapid and high-throughput screening of new antidepressant drugs. Timing the immobility time in the FST after an acute administration of AdipoRon allowed us to assess the kinetics of action, the dose-response effect and the route of administration (Fig. S2, SI-R1). A single *ip* or *per os* administration of AdipoRon produced a transient and dose-dependent reduction of the resignation-like behavior on the FST on different mouse models of depression. Furthermore, we showed that AdipoRon can cross the BBB and directly target the brain by activating intracellular signaling pathways (Fig. S3, SI-R2).

### Chronic AdipoRon treatment prevents neuroinflammation, restores the normal kynurenine pathway and increases serotonin turnover in the brain

**Fig. 3:**
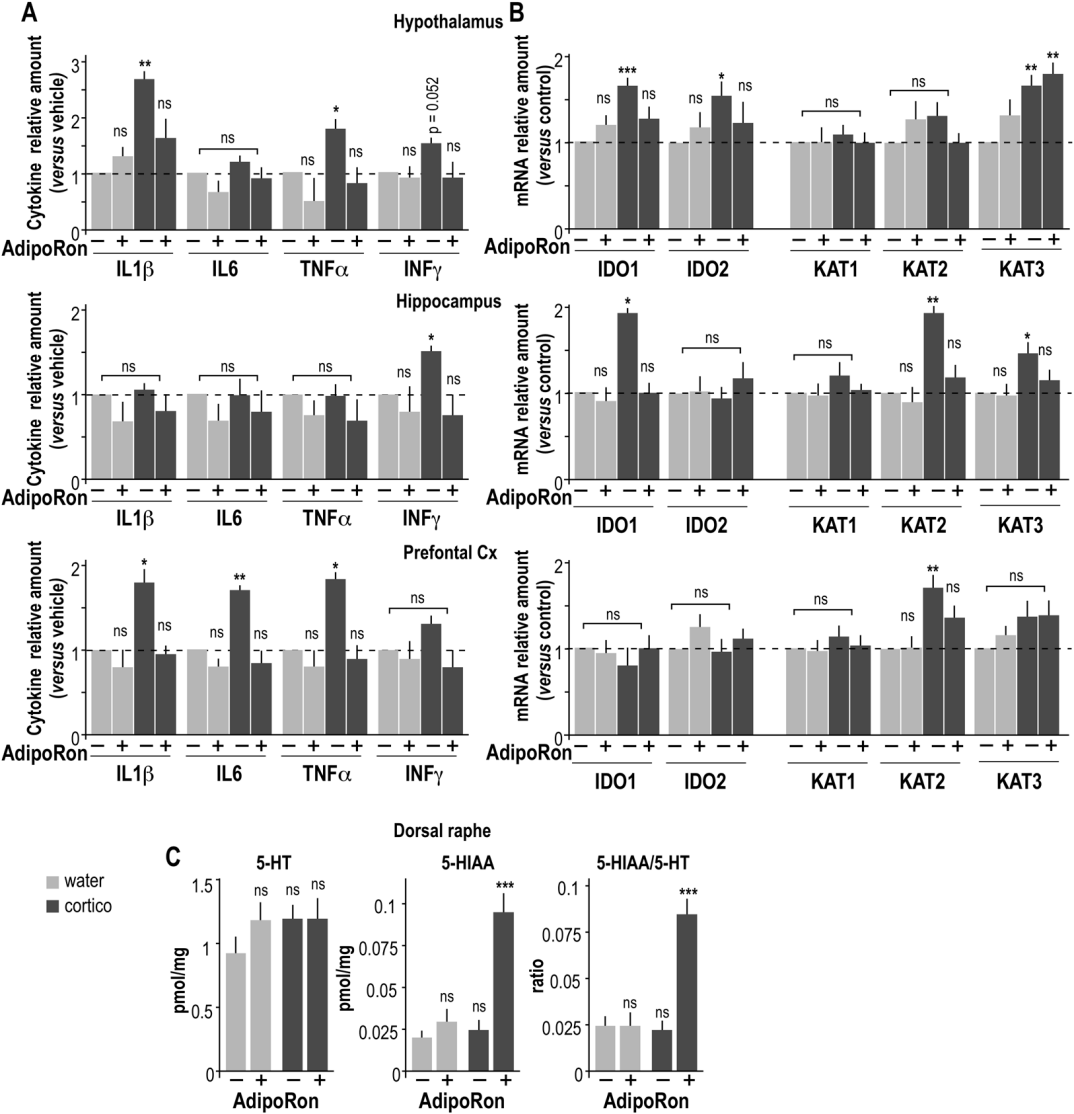
AdipoRon alleviates depression-related neuroinflammation and the Trp/L-Kyn pathway. **a** Proinflammatory cytokines IL1β, IL6, TNFα, and INFγ were measured from the hypothalamus, the hippocampus and the prefrontal cortex using a multiarray assay from control (light gray) and cortico-treated mice (dark gray) chronically treated by vehicle (−) or 1 mg/kg AdipoRon (+). **b** The expression of genes that encode enzymes involved in the Trp/L-Kyn pathway, i.e., IDO1, IDO2, KAT1, KAT2, and KAT3 was assessed by quantitative PCR in the hypothalamus, the hippocampus and the prefrontal cortex from control and cortico-treated mice ± AdipoRon administration. Data are expressed as relative amount (means data from control vehicle-treated mice taken as 1) are expressed as mean ± sem. **c** Concentration of 5-HT and its metabolite 5-HIAA assessed using HPLC analysis from the dorsal raphe of control (light) and cortico-treated mice (dark) ± 1 mg/kg of AdipoRon as described in SI-MM7. Histograms are means ± sem of 5-HT (left) and 5-HIAA (center) concentrations expressed in pmol/mg of total protein and the ratio 5-HIAA/5-HT (right). *N* = 8 *per* group; Kruskal–Wallis statistical analysis was followed by a Dunn’s statistical test for comparison with the vehicle control group. ns non-significant; *P < 0.05; **P < 0.01; ***P < 0.001

Neuroinflammation plays a crucial role in the pathogenesis of depression. To investigate whether AdipoRon displayed similar anti-inflammatory properties to ApN, the concentration of proinflammatory cytokines (IL1β, IL6, TNFα, IFNγ) in various brain regions involved in depression (i.e. hypothalamus, hippocampus, and prefrontal cortex) was measured using a multiplex assay (Fig. 3a). IL1β and TNFα concentrations were elevated in hypothalamus from cortico-treated mice compared to control, while in the hippocampus; only IFNγ was increased by cortico-treatment (Fig. 3a). The prefrontal cortex of cortico-treated mice showed increased levels of IL1β, IL6, and TNFα, but not IFNγ (Fig. 3a). In all brain regions, AdipoRon restored physiological concentrations of cytokines and thus abolished the cortico-induced neuroinflammation (Fig. 3a).

The expression levels of IDO and KAT mRNA were assessed by quantitative PCR in brain areas of interest (Fig. 3b). Chronic cortico-treatment significantly increased the level of expression of IDO1 and KAT3 in the hypothalamus, IDO1, KAT2, and KAT3 in the hippocampus, but only that of KAT2 in the prefrontal cortex (Fig. 3b). AdipoRon treatment restored the physiological levels of expression for both IDO and KAT in each brain region that was investigated in depressive-like mice (Fig. 3b).

The “serotonin hypothesis” postulates that diminished activity of the serotonin pathway plays a causal role in the pathophysiology of depression. Both AdipoR1 and AdipoR2 were expressed in the dorsal raphe, the largest serotonergic nucleus (Fig. S4). It was therefore of interest to determine if AdipoRon altered the levels of serotonin (5-HT) and its metabolite 5-hydroxyindoleacetic acid (5-HIAA) in the dorsal raphe from control and depressive-like mice. However, in dorsal raphe from depressive-like mice, AdipoRon increased the level of 5-HIAA without impacting that of 5-HT, resulting in a three-fold increase in the 5-HIAA/5-HT ratio (Fig. 3c). Chronic administration of AdipoRon increased the release and turnover of serotonin in the raphe nucleus from depressive-like mice (Fig. 3c).

A direct action of AdipoRon on serotonergic neurons of the dorsal raphe cannot be ruled out. To explore this hypothesis, patch-clamp experiments were conducted on brain slices in the whole-cell mode. In serotonergic neurons of the dorso-median raphe, AdipoRon led to an increase in action potential discharge frequency that was accompanied by a small depolarization (Fig. S4).

### Chronic AdipoRon treatment reverses the deleterious effect of long-term cortico-treatment on hippocampal neurogenesis

**Fig. 4:**
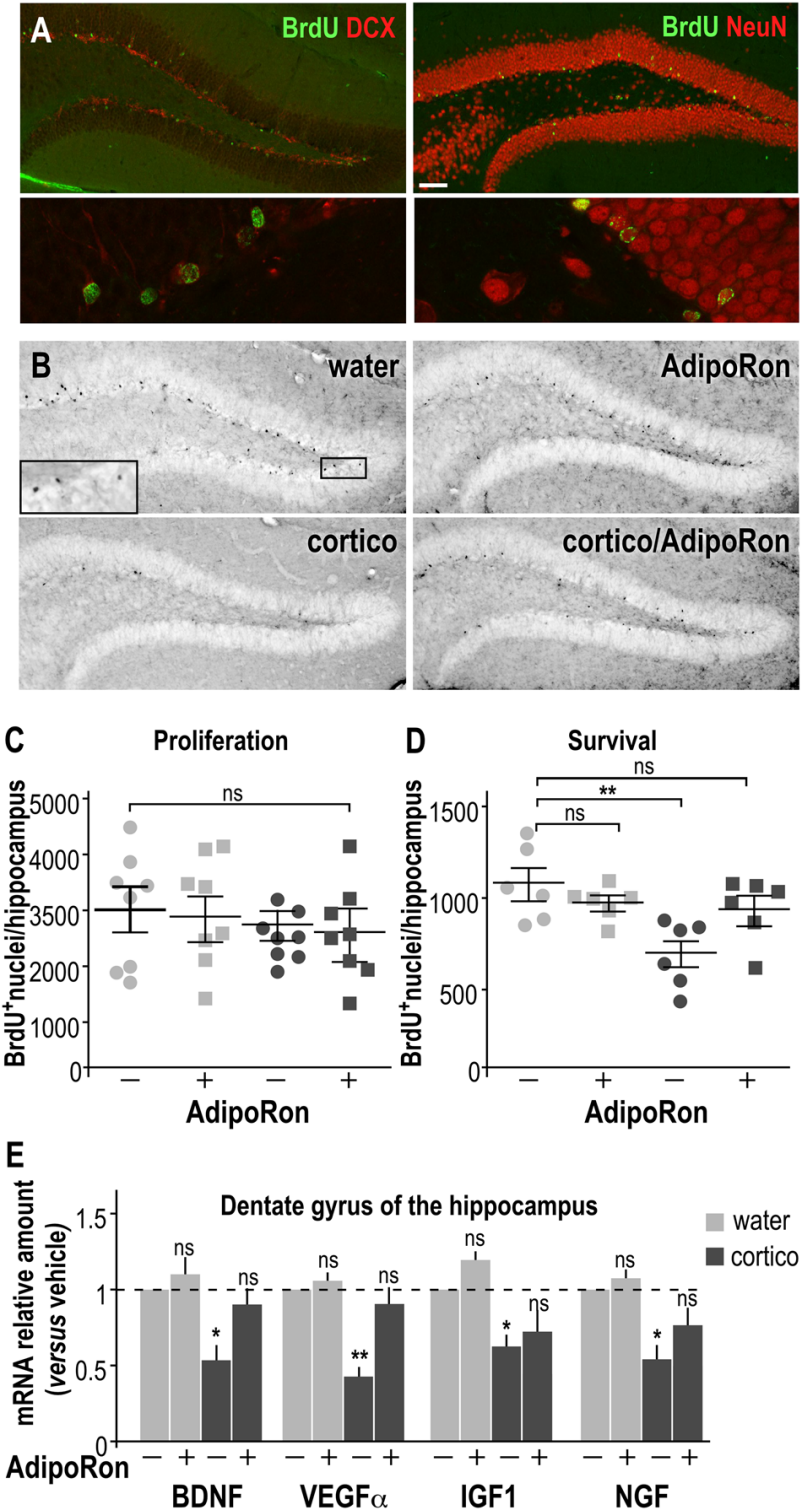
AdipoRon rescues neurogenesis in the dentate gyrus of the hippocampus of depressive-like mice. **a** Representative immunohistochemical images depicting co-staining BrdU-labeled nuclei (green) and DCX- (left, red) or NeuN-positive neurons (right, red). Upper panel: ×10 magnification bar, 100 μm; Lower panel: ×60 magnification bar, 10 μm. **b** Representative immunohistochemical images depicting BrdU-labeled nuclei (black, insert) of dentate gyrus slices from brains of vehicle-treated (upper) or chronically cortico-treated (lower) mice treated with AdipoRon (right) or vehicle (left). **c** BrdU (50 mg/kg of body weight) was injected three times, 3-h apart one day before sacrifice to examine the effects on neuronal proliferation after 7 weeks of cortico treatment ± AdipoRon (1 mg/kg/day). **d** BrdU was administered daily during the cortico treatment for 5 consecutive days prior the initiation of a 3-week period of treatment ± AdipoRon to assess for neuronal survival. Experiments were conducted as described in SI-MM3. Data represent the number of BrdU-positive nuclei counted in the dentate gyrus from the four groups of mice. Values plotted are mean ± sem; each symbol represents a mouse. **e** Dentate gyri were micro-dissected from hippocampus from long-term cortico-treated mice ± AdipoRon administration. The level of expression for genes involved in synaptic maturation, i.e., BDNF, VEGFα, IGF1, and NGF was determined by quantitative PCR. Histograms are means ± sem expressed as fold-change compared to vehicle control group. *N* = 8/group; Kruskal–Wallis followed by a Dunn’s post hoc test for comparison with the vehicle control group; ns non-significant, *P < 0.05, **P < 0.01

To further investigate the potential cellular mechanisms underlying the benefits of AdipoRon on depression-like behavior, we evaluated changes in adult hippocampal neurogenesis and neuronal survival (Fig. 4a, b). Neither cortico nor AdipoRon treatment modified the proliferation of neuronal precursors (i.e., assessed one-day post BrdU injection, Fig. 4c). However, long-term cortico administration significantly decreased the rate of survival of newborn hippocampal neurons, while AdipoRon reversed this deleterious effect (Fig. 4d). AdipoRon counteracted the adverse effects of cortico on the survival of hippocampal neurons without having any effects in control conditions (Fig. 4c, d). The expression of the genes encoding neurotrophic factors (namely BDNF, VEGFα, IGF1, and NGF) was down-regulated in the dentate gyrus of cortico-treated mice, AdipoRon restored physiological levels of expression of genes encoding these neurotrophic factors (Fig. 4e).

## Discussion

HPA axis dysfunction and sustained elevation of plasma glucocorticoids are involved in many of the physiological changes associated with metabolic syndrome and depression. Here we demonstrate that AdipoRon, a potent agonist of ApN receptors, efficiently reverses cortico-induced early onset of moderate obesity, metabolic syndrome, and depression-like behaviors in mice. Together, our study highlights the pivotal role of the adiponergic system in the development of these diseases and provides a novel and promising pharmacotherapeutic approach.

Previous studies have shown that mice deficient in ApN exhibit increased susceptibility to stress-induced depressive behaviors^3,22^. Intracerebral administration of exogenous ApN produces antidepressant-like effects in mice^22^ suggesting that ApN receptor agonists could exert similar effects. In agreement with this assumption, we demonstrate that AdipoRon exhibits antidepressant-like properties. AdipoRon is of particular interest because it was initially selected based on its ability to bind both adipoR1 and adipoR2 with similar affinities, thus globally activating the ApN-related intracellular pathways^24^. Furthermore, AdipoRon prevents obesity-related disorders in db/db mice^24^. As reliable readout, we performed a panel of behavioral tests aiming to assess a numerous aspects of the depression-like state, including anxiety (L&D), resignation (FST, LH), lower motivation (NSF), loss of pleasure (sucrose preference test), and social withdrawal^2,25–27^. In all of these paradigms, daily treatment with AdipoRon for 3-weeks successfully reversed the cortico-induced depression-like state in mice.

Childhood and adolescence are critical periods of development. During this period, hormonal and neuroendocrine disruptors are likely to have important implications for physiological and neurobehavioral functions in adulthood. During these phases, the HPA axis is maturing, as are tissues that respond to glucocorticoids, including adipose tissue and brain. It is likely that the continuous intake of glucocorticoids from the youngest age may lead to adulthood metabolic syndrome. Metabolic syndrome is defined as a cluster of disorders, including abdominal obesity, insulin resistance, and dyslipidemia. In this work, mice were chronically exposed to cortico from 5-weeks of age, for seven consecutive weeks. Cortico-treated mice exhibited variations in metabolic outcomes (body weight, and abdominal fat) compared to controls, suggesting the onset of metabolic syndrome. Interestingly, AdipoRon thwarts the cortico-induced effects, and prevents the increase of adiposity and body weight in this model. It should be noted that, some but not all of the indicators of metabolic syndrome were established at the adulthood. Indeed, while increase of body weight, abdominal fat, adipocyte size, hyperlipidemia, and hyperleptinemia was observed in cortico-treated mice, no systemic inflammation was observed. Yet, a chronic low-grade inflammatory state with production of proinflammatory cytokines such as IL1β, IL6, TNFα is the hallmark of metabolic disorders^9^. Conversely, in the experimental protocol described above, anti-inflammatory properties of cortico were still effective, as shown by decreased concentrations of TNFα and IL1β in plasma and WAT. The chemokine MCP1 controls the recruitment of monocytes from the blood stream across the vascular endothelium for routine immunological surveillance of tissues, and in response to inflammation and tissue injury^28^. The increased MCP1 production in WAT of cortico-treated mice could reflect the onset of WAT dysfunctions and thus portend future infiltration of macrophages and inflammation. Interestingly, AdipoRon limits MCP1 production, thus is likely to prevent or delay impairment of WAT function.

Adipokinemia is closely linked to waist circumference and abdominal adipose tissue distribution^29^. The leptin/ApN ratio is now considered to be an indicator of metabolic syndrome. It is more reliable than independent plasma adipokine concentrations and correlates positively with all metabolic symptom criteria^30–33^. In the present study, an elevated plasma leptin/ApN ratio was found in cortico-treated mice, compared to controls, suggesting the onset of obesity and metabolic syndrome.

To our knowledge, this is the first report showing the concomitant occurrence of moderate visceral obesity, metabolic syndrome, and anxio-depressive-like behaviors following cortico treatment. Our data suggest that dysfunction of the HPA axis early in life triggers parallel neurobehavioral and metabolic disorders, as opposed to a model in which one disorder (i.e., depression) is the consequence of the other (i.e., metabolic syndrome). To explore the mechanisms underlying the antidepressant actions of AdipoRon, we investigated the possible effects of AdipoRon on some depression-related neurochemical dysfunctions, i.e., neurogenesis, serotonergic neurotransmission, neuroinflammation, and the Trp/L-Kyn metabolic pathway. Although current depression research largely focuses on “the neurogenic hypothesis”, both neurogenesis-dependent and neurogenesis-independent pathways are likely to be involved in antidepressant-related actions^2^. In agreement with previous reports, we found that chronic cortico-treatment altered neuronal cell differentiation in adult hippocampus^2,3,34^. As demonstrated with other antidepressants, including fluoxetine^2,35^, AdipoRon efficiently counteracts the deleterious effect of long-term cortico-treatment on neurogenesis. However, a major difference between existing antidepressants and AdipoRon was revealed. Whereas chronic SSRI treatment, including fluoxetine, stimulates all stages of the neurogenesis process, i.e., proliferation, differentiation, and survival of neuronal cells in healthy control mice^2^, the effects of AdipoRon were exclusively observed in depressive-like mice. This suggests a significant difference in the SSRI and Adiporon mechanisms of action on adult neurogenesis. One can assume that AdipoRon acts by counteracting the deleterious cortico-induced pathways, rather than by directly stimulating neuronal survival and/or differentiation pathways. In agreement with this assumption, chronic cortico-treatment has been shown to potentiate the behavioral and neuroplastic effects of fluoxetine in C57BL/6 mice^36^.

Changes in the serotonin system elicited by chronic stress and/or the depression-like state might explain the effects that AdipoRon produced exclusively in depressive-like mice, but not in naive mice. The serotonergic neurotransmission strongly depends on the negative feedback function ascribed to 5-HT1A autoreceptors in the somatodendritic neuron of the dorsal raphe. Clinical studies have clearly established a crucial role of 5-HT1A autoreceptors in mood disorders and antidepressant treatments^37^. Long-term administration of cortico results in the desensitization of 5-HT1A autoreceptors and an antidepressant response^36,38^. Our data show an elevation of the 5-HIAA/5-HT ratio in the dorsal raphe of cortico-treated mice in response to AdipoRon is in favor of a greater enhancement of brain serotonergic neurotransmission and turnover. Finally, the direct effects of AdipoRon on 5-HT neurons should not be neglected, since acute application of AdipoRon on dorsal raphe slices increases serotonergic neuron firing.

The Trp/Kyn pathway is at the interface between inflammation and the serotonergic system, and has thus received increased attention in the pathophysiology of depression over the last decade^39–41^. Thus we investigated cortico-induced changes to the profiles of both proinflammatory cytokines and the expression of genes that encode Trp/L-Kyn pathway-related enzymes, namely KAT and IDO, in the brain regions involved in depression. Neuroinflammation manifested mainly in the prefrontal cortex and the hypothalamus of anxiodepressive-like mice. Increased levels of gene expression for enzymes involved in Trp catabolism were found in the prefrontal cortex, hippocampus and hypothalamus of anxio-depressive-like mice. These results suggest that cortico exposure leads to a reduction of essential amino acid availability (Trp and 5-HT) and/or an increase in neurotoxic metabolites as has previously been proposed^39^. Together, a long-lasting neuroinflammation profile and an alteration to the Trp/L-Kyn pathway could contribute to the emergence of anxio-depressive-like behaviors^42,43^. Interestingly, AdipoRon treatment reverses both phenotypes in cortico-treated mice without having a significant effect on control mice. Low-grade peripheral inflammation and the Trp/L-Kyn metabolic pathway are potential pathophysiological links between metabolic syndrome and depression^44^. In mice, plasma KynA/L-Kyn and L-Kyn/Trp ratios appear to be reliable indicators of cortico-induced behavioral and metabolic alterations as well as AdipoRon-induced recovery. Further epidemiologic studies are required to demonstrate the reliability and relevance of these ratios as predictive biomarkers for obesity-linked depression in humans.

The present study clearly demonstrates the antidepressant-like properties of AdipoRon in mice, likely through actions on multiple targets that are altered during mood disorders. AdipoRon fulfills the prerequisites for a medication, namely no or minor side effects, achievable doses in vivo, and a pharmacokinetic profile suitable for in vivo assessment. Although the use of AdipoRon as an antidepressant in humans will require further clinical investigations, AdipoRon appears to constitute a promising novel antidepressant.

## Electronic supplementary material

Fig S1

Fig S2

Fig S3

Fig S4

Supplementary Information rev
