# Supplementary figures and images for: Adiporon, an adiponectin receptor agonist acts as an antidepressant and metabolic regulator in a mouse model of depression
Source: Transl Psychiatry. 2018 Aug 16;8:159. doi: 10.1038/s41398-018-0210-y (PMC6095913; doi:10.1038/s41398-018-0210-y)

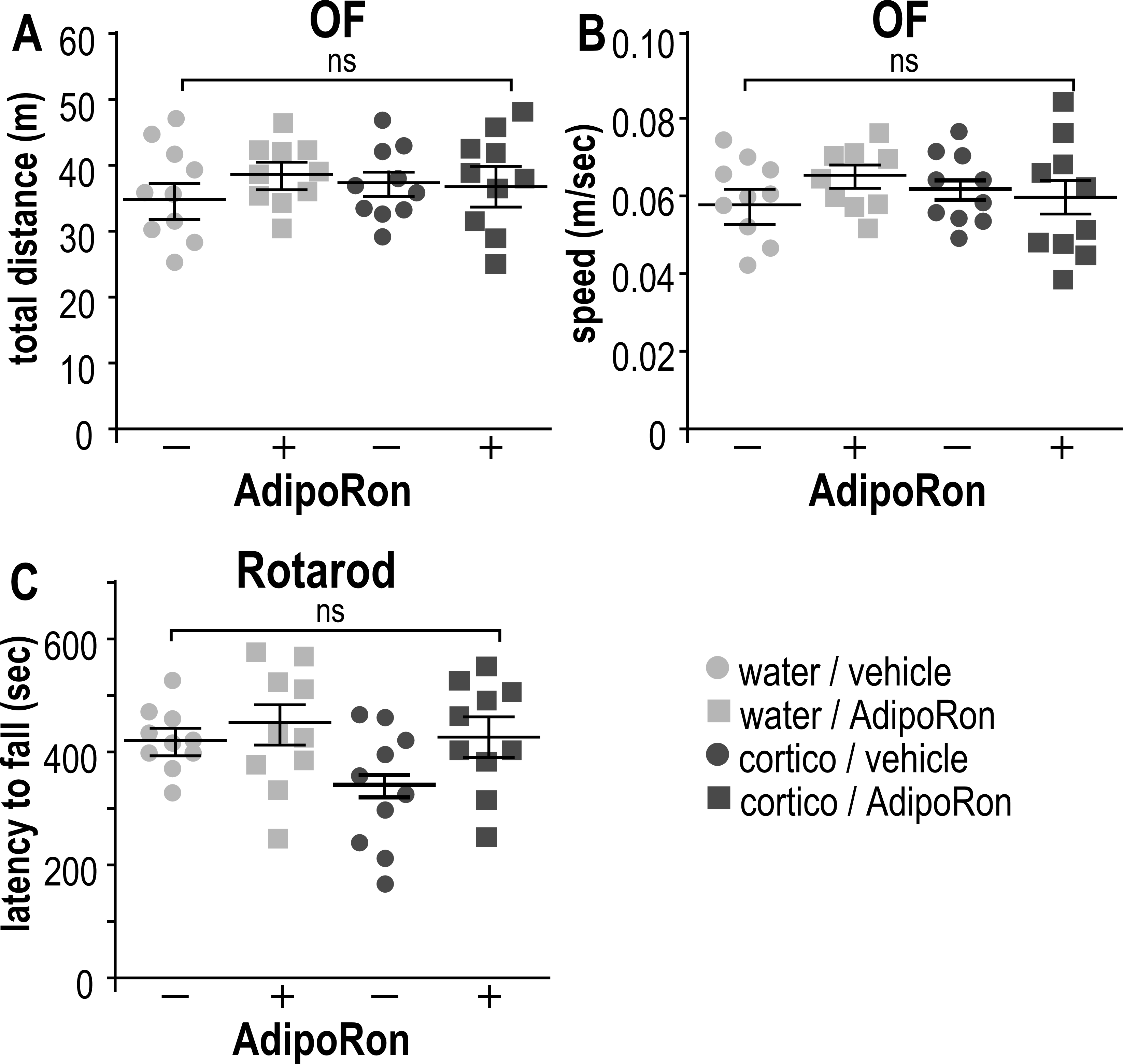

Supplement: Supplementary file 1 — Fig S1 [file 41398_2018_210_MOESM1_ESM.tif]

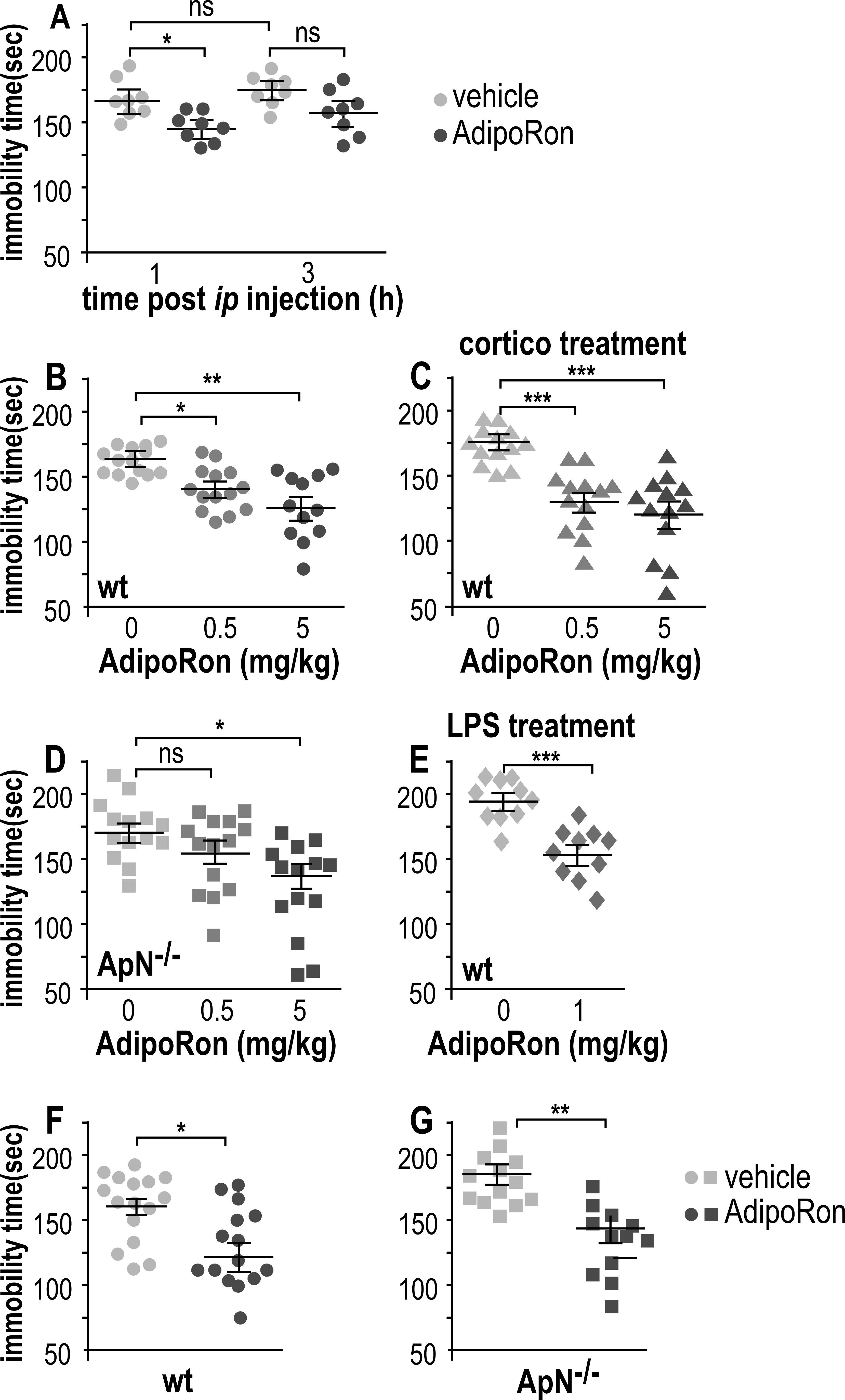

Supplement: Supplementary file 2 — Fig S2 [file 41398_2018_210_MOESM2_ESM.tif]

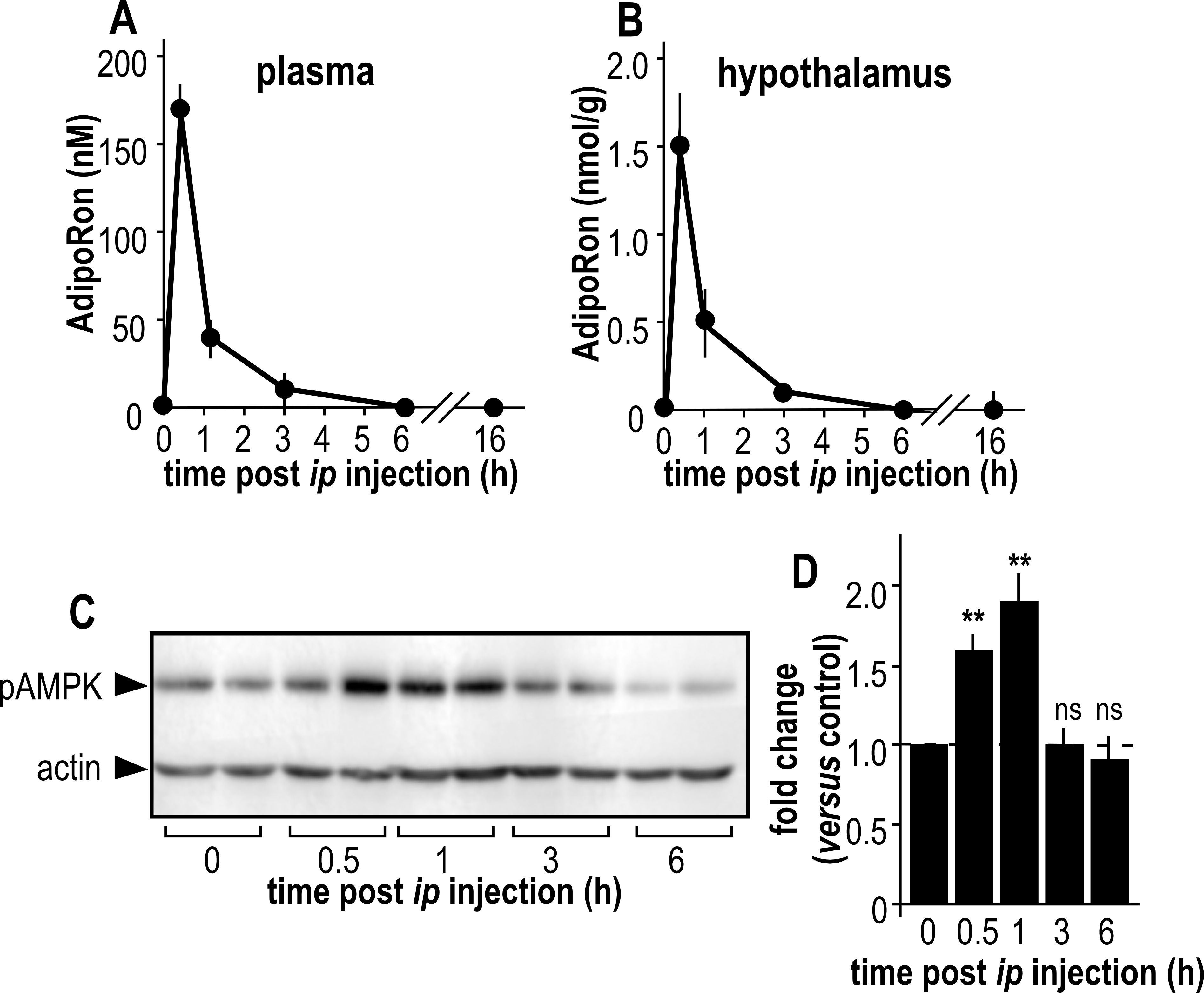

Supplement: Supplementary file 3 — Fig S3 [file 41398_2018_210_MOESM3_ESM.tif]

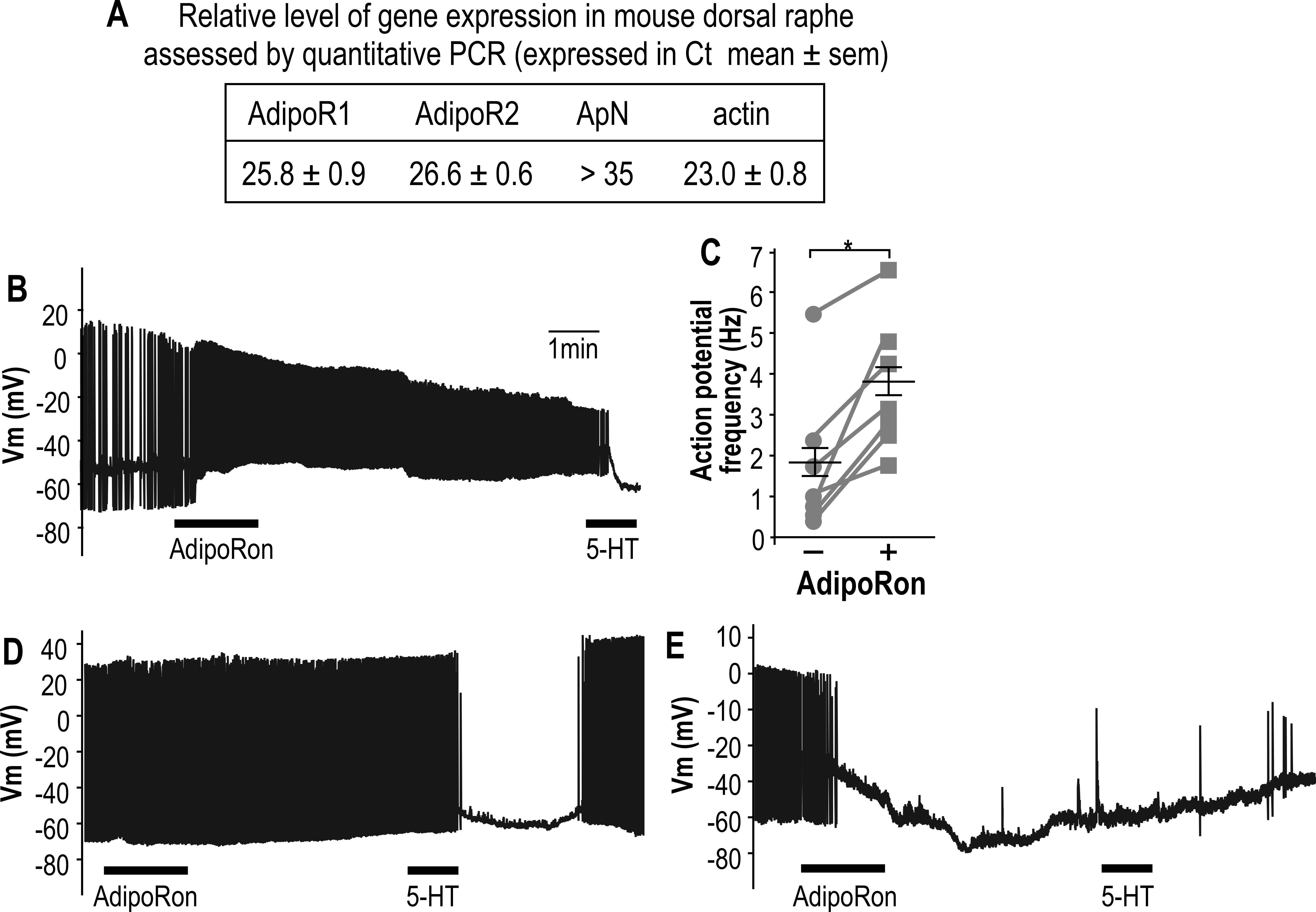

Supplement: Supplementary file 4 — Fig S4 [file 41398_2018_210_MOESM4_ESM.tif]
