# Supplementary material for: Adiporon, an adiponectin receptor agonist acts as an antidepressant and metabolic regulator in a mouse model of depression
Source: Transl Psychiatry. 2018 Aug 16;8:159. doi: 10.1038/s41398-018-0210-y (PMC6095913; doi:10.1038/s41398-018-0210-y)
Supplement: Supplementary file 5 — Supplementary Information rev [file 41398_2018_210_MOESM5_ESM.docx]

**Supplementary Information**

**Methods and Materials**

**SI-Materials-** AdipoRon, dimethylsulfoxyde (DMSO), lipopolysaccharide (LPS, from Escherichia Coli 0111:B4), bromodesoxyuridine (BrdU), paraformaldehyde (PFA), corticosterone (cortico), β-cyclodextrine (β-CD), Bradford reagent, EDTA, sodium deoxycholate, NP-40, tween 20, triton X100, protease cocktail, phosphatase inhibitors, bovine serum albumin (BSA) were purchased from Sigma-Aldrich (Saint-Quentin Fallavier, France).

**SI-MM1- *Acute treatment of AdipoRon in mouse models of acute sickness and depression-like behaviors.*** Three well-established mouse models of depression-like behavior have been used in the present study namely, **a-** long-term corticosterone (cortico) treatment in the drinking water of wt mice (2), **b-** ApN^-/-^ mice (25), and **c-** the systemic injection of LPS (42) as an acute sickness behavior model. For the acute sickness model, eight-week-old male mice were *ip* injected daily with AdipoRon or vehicle for twenty consecutive days. One day prior to behavioral testing, mice were *ip* injected with LPS (0.83 mg/kg in saline solution) or vehicle alone; the LPS dose was selected on the basis of its ability to induce the full spectrum of the acute sickness and depressive-like behaviors (45).

**SI-MM2- *Behavioral testing.*** Independent groups of control and cortico-treated mice were submitted to behavioral testing, or used for sample collection and immunohistochemistry (IHC) experiments as follows: groups #1 and #2 - IHC on brain sections from 1-day (#1) and 21-days post-BrdU injections (#2), group #3 - successively analyzed one day apart (OF, rotarod, L&D, FST), group #4 - successively analyzed two-days apart (sucrose preference, social interactions, NSF), group #5 - LH, and group #6 - IHC and biological analysis on dissected brain region (WAT and plasma).

Before each trial, the L&D, OF, rotarod, social interaction and LH devices were thoroughly cleaned with 70% ethanol and dried.

*Open-field test (OF)-* Locomotor activity was determined using an open-field (OF) arena. Mice were placed in the corner of the test apparatus (40Lx40Wx25H cm Plexiglas box) that was weakly illuminated (30 lux) for a 10 min session of free moving, and each entire session was videotaped. The automated analysis was performed using the ANY-maze software (ANY-maze^©^ version 4.6, Stoelting Co., USA) and resulted in a track record of the mouse movements in the arena, which allowed for evaluation of locomotor activity i.e. total distance traveled and mean speed.

*Rotarod-* Mice were placed on a rotating rod for a 5 min habituation period (10 rpm/min). The next day, the latency to fall, an index of the motor coordination, was recorded on an accelerating rod (from 10 to 40 rpm/min in 3.7 rpm/min increments).

*Light & Dark test (**L&D)-* The L&D place preference test was used to assess anxiety-like behavior. The test apparatus (40Lx30Wx25H cm) was divided into equal zones (i.e., light or dark zones) with a doorway connecting the two sides. The light zone was very bright (600 lux) while the dark zone was protected from light by an opaque lid. To initiate testing, mice were placed into the light side and activity was recorded for 5 min. The level of anxiety is inversely correlated to the time spent in the light zone.

*Forced Swimming test (FST)-* Mice were placed an inescapable water-filled (23°C ± 0.5) transparent tank (20 cm diameter, 40 cm deep) for 6 min, as previously described by Porsolt *et al.* (46). The immobility duration, was timed during the last four minutes of the test, as an indicator of resignation. A mouse was considered immobile when it remained floating with only slight movements to keep its head out of the water.

*Sucrose preference-* The sucrose preference test is based on the rodent interest in sweet solutions and used as an indicator of anhedonia. Mouse was placed in an individual cage and habituated to two water bottles for 24-hours. For the test, taking place during the 12 h dark session, the mouse had the choice to drink a 2% sucrose solution or tap water only. Sucrose preference was calculated as a percentage of the volume of sucrose intake over the total volume of fluid intake.

*Novelty Suppressed Feeding (NSF)-* NSF is a conflict test that elicits competing motivations: the drive to eat and the fear of venturing into the center of the brightly lit arena. The testing apparatus consisted of a plastic box, the floor of which was covered with approximately 2 cm of wooden bedding. Twenty hours prior to behavioral testing, all food was removed from the home cage. At the time of testing, a single pellet of food was placed on a piece of plastic at the center of the box, and the entire session was videotaped (i.e. 10 min period). The mouse was placed in a corner of the box and the latency to eat (defined as the mouse sitting on its haunches and biting the pellet) was timed. Immediately after the test, food consumption was measured for 30-min as a control for potential feeding differences.

*Social interaction test****-*** The social testing apparatus was a rectangular, three-chambered box with clear Plexiglas walls, and small circular openings (3.5 cm in diameter) that allowed for access to each chamber (25Lx15Wx20H cm). Three interconnected chambers were separated by manually-operated sliding doors. The test mouse was first placed in the middle chamber and allowed to explore the entire apparatus for a 5 min habituation period. At the end of the session, the test mouse was confined to the central chamber for 1 min by closure of the doorways between the two side chambers. A wire cup-like container was placed in the corner of each chamber; one was empty (*right chamber*), while the second (*left* *chamber*) contained an unfamiliar mouse with same genotype, sex and age range. Both doors to the side chambers were then unblocked and the test mouse was allowed to freely explore the entire test box for a 5 min period. The time spent in the right (*empty*) and left (*stranger mouse*) chamber was timed allowing for assessment of the interaction with an inanimate object *versus* sociability (47).

*Learned Helplessness (LH)-* The tested mouse was placed into a shock chamber and exposed to inescapable shocks comprised of 360 scrambled footshocks (intensity: 0.3 mA) with a 2 sec duration and an interval-episode of 10 sec, amounting to total session duration of 60 min. The shock procedure was repeated for four consecutive days. On the fifth day, learned helplessness was assessed by testing shuttle box performance. Each trial started with a 0.3 mA footshock with a maximum duration of 24 sec. The inter-trial interval was 60 sec. The following behavioral reactions were defined: “escape” as shuttling to the other compartment in reaction to the electric shock, and “failure” when no attempt to escape was made. Furthermore, the “*escape latency*” parameter was defined and recorded as the time needed to shuttle into the other compartment after onset of footshocks. The mice that failed to escape were excluded from the escape latency measurement. Total time of testing for helplessness lasted about 20-24 min, the exact time period depended on the animal’s ability to learn the paradigm and to respond. Control animals underwent the same handling and contextual procedures without receiving the footshocks.

**SI-MM3- *Hippocampal neurogenesis and neuronal survival.*** Serial coronal sections of the brains were cut (40 µm) throughout the hippocampus (from bregma -1.34 to -3.52) on a vibratome (Leica). Every fifth section (eight in total) from throughout the hippocampus was processed for BrdU immunohistochemistry as follows. Free-floating sections were first incubated for 1 hour at room temperature in PBS containing 2.5% horse serum then overnight at 4°C with a mouse anti-BrdU antibody (1/7500; BD Pharmingen #555627) and finally for 2 hours in biotin-conjugated species-specific secondary antibodies (1:400; Vector Laboratories, CliniSciences, Nanterre, France) followed by a peroxidase-avidin complex solution according to the manufacturer's protocol. The peroxidase activity of immune complexes was visualized with DAB staining using the VectaStain ABC kit (Vector Laboratories). BrdU-labeled cells in the subgranular and granule cell layers were counted in each section at 40x magnification under a light microscope (Olympus). The number of BrdU^+^ cells *per* eight slices along the rostro-caudal axis was multiplied by five to obtain the total number of cells within the entire dentate gyrus.

Alternatively, mouse free-floating sections were incubated with mAb anti-BrDu (1/7500) combined with rabbit pAb anti-NeuN (1/1000, Abcam #Ab177487) or anti-doublecortin (DCX; 1/500 Santa Cruz Biotechnology #SC8066) ; secondary donkey anti-mouse or anti-rabbit mAb coupled to AlexaFluor 488 (Invitrogen) were used for double immunofluorescence staining.

**SI-MM4- Quantitative reverse transcription (RT)-PCR-** Total RNA was isolated from frozen brain tissues with Trizol (Merck, France) according to the manufacturer instructions. 2 μg of total RNA was reverse transcribed using the Superscript III synthesis kit (Invitrogen, ThermoFisher, Courtaboeuf, France) then treated with RNase-free DNase (Invitrogen) for 30 min at 37°C. Diluted reactions were analyzed with SYBR green PCR reagents and a sequence detector (Rotor-gene 6000, Corbett Research, Qiagen). mRNA levels of mouse genes of interest were normalized to actin. The quality of the PCR was confirmed by control reactions without RT, slope of standard curves and dissociation curves of products. The primers used to evaluate the level of expression of various mouse genes were purchased from Qiagen (Valencia, CA, USA) and Eurogentec (Angers, France). The “comparative Ct” method and standard deviations were calculated according to User Bulletin #2 from Applied Biosystems (Section 7 ; <http://www3.appliedbiosystems>.com/cms/groups/mcb_support/documents/generaldocuments/cms_ 040980.pdf).

The following primers were purchased from Qiagen (Valencia, CA, USA): ApN (QT01048047), BDNF (QT01545348), VEGFα QT00160769), IGF1 (QT00154469), NGF (QT00093464), TDO2 (QT00150409), CRF (QT00293489), GR (QT00160349), actin (QT01136772). The following primers were from Eurogentec (Angers, France): AdipoR1 forward 5’-CTTCTACTGCTCCCCACAGC-3’ and reverse 5’-GACAAAGCCCTCAGCGATAG-3’; AdipoR2 forward 5’-AGCCTATCTGCCCTATGGTG-3’ and reverse 5’-CTGTGTGCTGGGCATTGCAG-3’; IDO1 forward 5’-CAAAGCAATCCCCACTGTATCC-3’ and reverse 5’-ACAAAGTCACGCATCCTCTTAAA-3’; IDO2 forward 5’-CCTCATCCCTCCTTCCTTTC-3’ and reverse 5’-GGAGCAATTGCCTGGTATGT-3’ ; TDO1 forward 5’-AACATGCTCAAGGTGATAGCTC-3’ and reverse 5’-GAACCGAGAACTGCTGTACCA-3’; KAT1 forward 5’-CGAAGGCTGGAAGGGATCG-3’ and reverse 5’-GCGGTGAGAAGTCAGGGAA-3’; KAT2 forward 5’-ATGAATTACTCACGGTTCCTCAC-3’ and reverse 5’-AACATGCTCGGGTTTGGAGAT-3’; KAT3 forward 5’-TTCAAAAACGCCAAACGAATCG-3’ and reverse 5’-GATGACCAAAGCCTCTTGTGT-3’.

**SI-MM5- *Acute brain slices and whole-cell patch-clamp recordings.*** Mice were deeply anesthetized with halothane, decapitated and the brains were immediately placed into ice-cold gassed medium (95% O_2_/5% CO_2_) containing (in mM): 125 NaCl, 2.5 KCl, 1 MgCl_2_, 0.4 CaCl_2_, 1.25 NaH_2_PO_4_, 26 NaHCO_3_ and 25 glucose. Coronal slices at the level of the fourth ventricle (350μM thick) were cut with an HM650V vibratome (Microm, Walldorf, Germany) and placed in a holding chamber at 34°C for 1 h. Slices were then transferred at room temperature in Phosphate Bicarbonate Buffer Saline (PBBS) composed of (in mM): 125 NaCl, 2.5 KCl, 1 MgCl_2_, 2 CaCl_2_, 1.25 NaH_2_P0_4_, 26 NaHC0_3_ and 25 glucose, pH 7.4 when bubbled with 95% O_2_ / 5% CO_2_.

Brain slices containing the raphe nucleus were placed under a Nomarski microscope (Zeiss, France) equipped with an infrared video camera (Axiocam, Zeiss, France) in a recording chamber superfused at a flow rate of 1 ml/min with oxygenated PBBS (2 mM CaCl_2_). Recordings from median and dorsal raphe nucleus neurons were made at room temperature (25 ± 2°C) using an Axopatch 200B (Axon Instruments, USA). Patch clamp pipettes made from borosilicate glass capillary (Hilgenberg, Germany) had a resistance of 3-8 MΩ when filled with the internal solution containing (mM): 130 K gluconate, 1 MgCl_2_, 0.3 CaCl_2_, 1 EGTA, 4 Mg_2_ATP, 0.4 Na_3_GTP, 10 Hepes, pH 7.3. Neurons were first patch-clamped in the cell-attached mode to record spontaneous action potential firing and then in the whole-cell configuration in current clamp. Values of access resistance ranged from 12 to 20 MΩ and were not compensated. Measurements were made 2-3 min after obtaining the whole-cell to ensure dialysis. Cell capacitance and resistance were measured by voltage clamp using the pClamp Clampex software by applying 5 mV voltage steps. Solutions were applied in the bath. Current-clamp recordings were made in the IClamp mode of the Axopatch 200B. 5-HT neurons from dorsal raphe were identified using the following criteria: a slow (0.5–2.5 Hz) and regular firing rate, a long-duration action potential (48) and a large hyperpolarization of membrane potential in response to the application of 5-HT at the end of the recording (presence of 5HT1a auto-receptors).

Voltage-clamp experiments were performed with a KCl solution of the following composition (mM): 390 KCl, 5 MgCl_2_, 1 CaCl_2_, 10 Hepes, 10 EGTA, 4 Na_2_ATP, 0.4 Na_3_GTP (pH 7.3, 300 mOsm). Data were digitized at 5-10 kHz using a Digidata interface coupled to a microcomputer running pClamp 9 (Axon Instruments, USA) and digitally filtered at 1-3 kHz. Average data were expressed as mean ± sem, n = number of neurons, N = number of mice.

**SI-MM6- *Plasma determination of AdipoRon and tryptophan metabolites.*** Plasma levels of AdipoRon, serotonin (5-HT), L-kynurenin (L-Kyn), tryptophan (Trp), kynurenin acid (KynA) were determined as follows: 25 µl of plasma was deproteinated with 150 µl of cold methanol, incubated on ice for 20 min then centrifuged at 10,000 x g for 10 min at 4°C; supernatants were dried under speed vacuum centrifuge then resuspended in 50 µl of a 90% water, 10% acetonitrile, 0.1% formic acid mixture. Determination of AdipoRon and metabolite amounts were performed in triplicate (5 µl of sample) and determined by liquid chromatography (HPLC, using a Dionex U3000 RSCL Instrument) coupled to an MS-MS equipped with an ESI source. The separation for Adiporon was performed using a Nucleodur C18 gravity-SB column (150 mm x2 mm i.d. x 1.8 µm) from Macherey-Nagel operated at a 350µl/min flow rate. The following elution program based on water (solvent A) and acetonitrile (solvent B), both containing 0.1% formic acid (v/v): 0 min 2%B, 15 min 34%B, 20 min 95%B. The separation conditions for 5-HT, L-Kyn, KynA and Trp were performed using an Accucore PFP colum n(150 mm x 2.1 mm i.d. x 2.6 µm) from Thermo Scientific operated at a 400µl/min flow rate. The following elution program based on water (solvent A) and acetonitrile (solvent B) both containing 0.1% formic acid (v/v): 0 min 0%B, 2 min 0%B, 8 min 6%B and 13 min 40%B. The Q-exactive *plus* spectrometer was completely controlled by the XCalibur software and operated in electrospray positive mode. Typical ESI conditions were as follows: electrospray voltage 3.5 kV; capillary temperature 320°C, probe temperature 350°C, sheath gas flow 40 U and auxiliary gas 12 U. It was operated in PRM (Parallel Reaction Monitoring) mode. The quadrupole was set to transmit the protonated molecular ions at m/z 429.21 for AdipoRon, m/z 209,09 for L-Kyn, m/z 205.09 for Trp, m/z 190.05 for KynA and m/z177.10 for 5-HT. The protonated molecular ions were fragmented by HCD (High Collision Dissociation) as shown in the table 1. Data acquisition and processing were carried out using XCalibur Quan-Browser software version 3.0.

***Transitions from parallel reaction monitoring analysis***

| Compound Name  *m/z value* | Collision Energy (V) | Fragment m/z value |
| --- | --- | --- |
| AdipoRon  *429.2172* | 27 | 322.143  174.127 |
| L-Kyn  *209.0921* | 15 | 174.054  146.059 |
| Trp  *205.0971* | 35 | 188.0705  146.0600 |
| KynA  *190.0498* | 15 | 162.054 |
| 5-HT  *177.1021* | 10 | 160.0757  132.0809 |

**SI-MM7- *5-HT and 5-HIAA quantification in brain regions.*** The concentrations of serotonin (5-HT) and its metabolite 5-hydroxy-indol-acetic acid (5-HIAA) were quantified by HPLC. Dorsal raphe were homogenized by sonication in 0.1 N HCl and then centrifuged at 3 000 rpm for 10 min at 4°C. The supernatant was then filtered by centrifugation through a Nanosep 3K device (Pall Co Sigma) at 15 000 rpm for 30 min at 4°C, and then stored at -80 °C. Diluted samples (10 µl, 1:20 in 0.1 N HCl) were injected into a reverse-phase HPLC column (Thermo HR-80 RP-18 column; 80 x 4.6 mm; 3 µm). The mobile phase for the 5-HT/5-HIAA analysis was: 0.1 M NaH_2_PO4, 0.1 mM EDTA, 2.75 mM octane sulfonic acid, 0.25 mM triethylamine, 15% methanol, 5% acetonitrile, pH 2.9 delivered at 0.7 ml/min by an ESA-580 pump. An ESA coulometric detector (ESA Coulochem III connected to a graphite-electrodes analytical cell, ESA 5014B) was used for electrochemical detection. The conditioning electrode was set at –0.175 mV and the detecting electrode was set at + 0.175 mV.

**SI-MM8- *Immunoblotting.*** Total proteins were extracted from mouse hypothalamus samples by homogenization in lysis buffer (50 mM Tris-HCl, pH 7.5, 150 mM NaCl, 5 mM EDTA containing 0.5% Triton X-100 and 0.5% sodium deoxycholate with a cocktail of protease and phosphatase inhibitors). Equal amounts of total proteins (50 µg), as determined by the Bradford method (BioRad), were separated by 12% SDS-PAGE, then transferred to a nitrocellulose membrane (Schleicher & Schuell, Dassel, Germany). Blots were incubated in PBS 0.1% Tween 20, 2% BSA with mAb directed against phospho-AMPKα (Thr172; 1/1000; Cell Signaling #2535) or total AMPK (1/1000; Cell Signaling #2532), then with the appropriate HRP-coupled secondary mAb. Blots were developed using an enhanced chemoluminescence system (Immobilon, Merck Millipore) with a Fusion detector (Vilber). To correct for any loading artifact, blots were re-probed with anti-actin pAb (Abcam #Ab8227). Densitometry analyses were performed with the “National Institutes of Health” Image software^TM^ on the immuno-positive bands.

**Results**

**SI-R1-** Mice were *ip* injected with AdipoRon (1 mg/kg) or vehicle alone, then submitted to the FST 1 h or 3 h later. Compared to the vehicle, AdipoRon decreased the immobility time of wt mice when measured 1 h post-injection, whereas no significant effect was observed 3 h post-injection (Fig. S2A). Dose-response experiments were conducted on wt (Fig. S1B) and depression-like mouse models, namely cortico-treated (Fig. S2C), ApN^-/-^ (Fig. S2D) and LPS-treated mice (Fig. S2D). One hour post-injection, 0.5 mg/kg of AdipoRon significantly reduced the immobility time of wt and cortico-treated mice (Fig. S2B, C). Doses of 5 mg/kg and 1 mg/kg of AdipoRon were required to produce an effect in ApN^-/-^ (Fig. S2D) and LPS-treated mice (Fig. S2E). An alternative route of administration, i.e. *per os*, was then investigated. AdipoRon administered *per os* significantly reduced the immobility duration in the FST of both ApN^-/-^ and wt mice.

**SI-R2-** The possibility that AdipoRon could cross the blood-brain barrier and trigger activation of intracellular signaling pathways was investigated as follows. Plasma samples and brain regions of interest (hypothalamus, and hippocampus) were collected from mice at different time points after *ip* injection of 1 mg/kg AdipoRon. The kinetics of BBB crossing and the recovery rate of AdipoRon in plasma and brain samples were determined by HPLC coupled to MS/MS analysis. The highest amount of AdipoRon was measured 30 min post *ip* injection in plasma (Fig. S3A), the hypothalamus (Fig. S3B) and the hippocampus (1.41 nmol/g ± 0.32) and then it decreased until it was barely detectable 3 hours post injection.

AdipoRon binds to ApN receptors activating various intracellular pathways, including AMPK phosphorylation (27). Immunoblot experiments were performed at different time points post *ip* injection of AdipoRon to determine the rate of AMPK phosphorylation within the hypothalamus. Interestingly, AdipoRon increased the level of phospho-AMPK 30 min and 1 hour post *ip* injection. These effects were no longer observable 3 hours post injection (Fig. 3D,E). We showed that AdipoRon can cross the BBB and directly target the brain by activating intracellular signaling pathways.

**Figure Legends**

**Figure S1-** **Absence of AdipoRon effects on locomotor and motor coordination.** Locomotor and exploratory activities (**A, B**) and motor coordination (**C**) of wt mice chronically treated with cortico (*dark gray*) or vehicle (*light gray*) with (+) and without (-) AdipoRon administration (*ip*, 1 mg/kg) were recorded in the open-field (OF) for 10 minutes (i.e. total distance **(A)** and mean speed (**B**) and in the rotarod (**C**) respectively, as described in the SI-MM2.

**Figure S2: A.** **Assessment of the antidepressant-like effect of an acute injection of AdipoRon using the FST. A.** Ten-week-old male wt mice were *ip* injected with Adiporon (1 mg/kg, *dark gray*) or vehicle alone (*light gray*) one and three hours prior to the FST, which was performed as described in the SI-MM2. Untreated male wt mice (**B**), long-term treated mice with cortico in the drinking water (**C**) and ApN^-/-^ male mice were *ip* injected with the indicated concentration of AdipoRon (0.5 and 5 mg/kg) or vehicle alone (0) one hour prior the FST. **E.** Wt male mice were *ip* injected with LPS (0.83 mg/kg). 16 hours later, mice were *ip* injected with AdipoRon (1 mg/kg) or vehicle alone prior to the FST. **F, G**. AdipoRon (1 mg/kg) or vehicle alone was administered *per os* to wt (**F)** and ApN^-/-^ mice (**G**) one-hour prior the FST. The immobility time (in sec) was measured during the last four minutes of the six-minute test. Data are plotted as mean ± sem; each symbol represents a mouse; Kruskal-Wallis was followed by a Dunn's multiple comparison test (**A, B, C, D**). The Mann-Whitney statistical analysis was performed for comparison between two groups (**E, F, G**). ns, non-significant ; **P* < 0.05, ***P* < 0.01 ; ****P* < 0.001.

**Figure S3-** **AdipoRon crosses the BBB and targets the brain**. The concentrations of AdipoRon were measured in plasma (**A**), and hypothalamus (**B**) by HPLC-coupled to MS-MS analysis as described in SI-MM6 at different time points post *ip* injection of 1 mg/kg AdipoRon. Data are mean ± sem, area expressed in nM for the plasma and in nmol/mg of total protein for each region of the brain; N = 6 mice *per* group. **C.** Representative western blot showing the phosphorylation kinetics of AMPK in the hypothalamus from vehicle- (0) or AdipoRon-treated mice 30 min (0.5), 1 h, 3 h or 6 h post *ip* injection as assessed by western blotting (SI-MM8). **D.** Histogram showing the quantification of pAMPK levels expressed as fold-change compared to the vehicle-injected group. Data are expressed as mean ± sem of the four independent experiments, N=2 *per* time point *per* experiment. Kruskal-Wallis was followed by a Dunn’s post-hoc test for comparison with the vehicle control group; ns, non-significant; ***P* < 0.01.

**Figure S4: Adiporon modulates serotonergic neuron activity** **and 5-HT turnover in dorsal raphe**. **A.** Relative level of expression of ApN and ApN receptors in dorsal raphe from wt mice assessed by quantitative PCR (expressed in means Ct ± sem; N=4). **B.** Acute exposure of AdipoRon on dorsal raphe slices increased the frequency of discharge in action potentials and depolarized a presumed serotonergic neuron, which was characterized by its low frequency, regular pattern of discharge, and its hyperpolarizing response to an application of 5-HT at the end of the recording. Experiments were conducted as described in SI-MM5. **C.** The action potential frequency was measured for each recording using the clampfit threshold search method at one min intervals before and during application of AdipoRon (10 μM). Data are expressed as mean ± sem; each symbol represents a neuron; Mann-Whitney statistical analysis was performed; **P* < 0.05. **D.** AdipoRon had no effect on the action potential discharge in a presumed serotonergic neuron. **E.** AdipoRon hyperpolarized a presumed non-5-HT neuron, which was characterized by its lack of response to a 5-HT application at the end of the recording.
